# Effect of hydroalcoholic *Allium atroviolaceum* L. on the pathology of testicular tissue in cyclophosphamide-treated mice

**DOI:** 10.37796/2211-8039.1026

**Published:** 2020-09-01

**Authors:** Mehrdad Shahrani, Shirin Asgharian, Alireza Hosseini, Elham Bijad, Maryam Anjomshoa, Ayoob Rostamzadeh, Najmeh Asgharzadeh, Zahra Lorigooini

**Affiliations:** a Medical Plants Research Center, Basic Health Sciences Institute, Shahrekord University of Medical Sciences, Shahrekord, Iran; b Department of Embryology and Histology, Faculty of Medicine, Shahrekord University of Medical Sciences, Shahrekord, Iran; c Department of Anatomy and Neuroscience, Faculty of Medicine, Shahrekord University of Medical Sciences, Shahrekord, Iran

**Keywords:** Cyclophosphamide, *Allium atroviolaceum*, Pathology, Testicular tissue

## Abstract

**Background:**

The most important side effects of Cyclophosphamide, as an anticancer broad-spectrum drug, are the negative effects on the reproduction and fertility because of oxidative stress. Considering the antioxidant properties of medicinal plants, especially those of the *Allium* genus, this paper studied the effect of hydroalcoholic extract of *Allium atroviolaceum* L. on the pathology of testicular tissue in CP-treated mice.

**Methods:**

Groups of this experimental study consisted of normal saline recipients; three groups receiving *A*. *atroviolaceum* extract at 50, 100, 200 mg/kg; three groups receiving *A. atroviolaceum* extract at 50, 100, and 200 mg/g and 6.6 mg/kg of Cyclophosphamide; and a group given Cyclophosphamide at 1.6 mg/kg. All injections were performed intra-peritoneally. After 30 days, the testicular histological profile as well as the number of spermatozoa, the number of primary and round spermatocytes, and the number of spermatogonia were investigated.

**Results:**

Cyclophosphamide treatment significantly reduced the lumen diameter, the seminiferous tubule diameter, the epithelial thickness, as well as decreased the quantity of spermatozoa and round and primary spermatocytes compared to the control group. Cyclophosphamide groups treated with *A*. *atroviolaceum* extract at 50, 100 and 200 mg/kg in a significant manner improved these variables (*P* < 0.001).

**Conclusion:**

*A. atroviolaceum* extract can significantly improve Cyclophosphamide-induced toxicity and pathological process on testicular tissue. It seems that this plant, with high antioxidant capacity, can be considered a complementary therapy for Cyclophosphamide to prevent undesirable effects on the reproductive system.

## 1. Background

The *Allium* genus plants belong to the *Alliacea* family. The genus Allium includes approximately 750 plant species [1]. These herbs are rich in flavonoids, saponins, sapogenins, and vaporizable sulfur compounds. These compounds control the characteristics of these plants, including their spicy smell and taste. These compounds are unstable and are easily converted to other compounds [2]. Moreover, the antioxidant effects of various species of this genus have also been proven [3-5]. Plants of this genus have been used as food in the culture of some countries for centuries, and also as preventive and therapeutic drug compounds [6, 7]. It has been shown that the plants of this genus are effective on cardiovascular disease, tumor growth, and aging process. It seems all of these diseases are associated with the effects of free radicals [8, 9]. Infertility involves 15% of couples all over the world. The factors of infertility in men are responsible for about 40%-50% of all infertility cases [10]. The factors of male reason for infertility are obstruction, varicocele, infection, and exposure to toxins, rays, and certain drugs [11, 12]. Cyclophosphamide (CP) is one of these drugs that has been used extensively in recent decades [13-15].

This drug, despite its extensive impact on various types of cancers has many adverse effects including reproductive toxicity[16]. It has been shown that oligospermia, azoospermia, as well as histological and biochemical changes are induced in testicular tissue and epididymis by CP[17, 18]. CP can lead to damage by degrading regenerative reactions in tissues and developing oxidative stress[16].

Due to these side effects, as well as the mechanism of their occurrence, complementary therapies, especially the usage of medical plants for the cure of different diseases, including infertility, have become widespread. Therefore, further studies are needed in this regard [19-22].

The usage of medical plants to increase fertility and also to cure certain conditions, such as hormonal imbalance, impotence (sexual weakness), oligospermia, sperm movement, prostate inflammation, and varicocele, have long been considered. On the other hand, the positive effect of certain plants of the *Allium* genus has already been studied on infertility.

*A. cepa* and *A. sativum* are among the species whose effect on fertility and the reproductive system has been shown[23, 24]. Black-violet Leek, botanically referred to as *Allium atroviolaceum* L., is another species of the Alliaceae family and the *Allium* genus [25].

Considering the antioxidant effect of *A*. *atroviolaceum*, this investigation was carried out to examine the effect of this plant on the pathology of testicular tissue in CP-treated mice. This choice was due to its availability and the effects of this plant on improving men's fertility.

Due to the availability of this plant to the public usage, determining its effects on the reproductive system could lead studies to commercialize production.

## 2. Methods

### 2.1. Plant material and Extraction

*Allium atroviolaceum*, the wild leek with broadleaf, is a plant species endemic to Iran and widely used as a food source. The aerial part of early stage growing of *A*. *atroviolaceum* was gathered in the west of Iran from Sabzkooh mountain in May 2014 and then identified by a skilled botanist (Shirmardi Hamzeh Ali, PhD., Research Center of Agriculture and Natural Resources, P.O. Box 415, Shahrekord, Iran); a herbarium voucher (801) was assigned to the prepared plant specimen at the Medical Plants Research Center of Shahrekord University of Medical Sciences. Then, the plant was dehydrated by freeze dryer and ground by an electric mill. The extraction was carried out by maceration method with 70% ethanol. After 72 h, the filtrated extract was concentrated with a rotary evaporator and then dehydrated in an incubator at 37 °C [26].

### 2.2. Animals

64 male NMRI mice weighing 25-30 g and 7 weeks of age, were bought from the Tehran Razi Institute, and kept in the animal house of Shahrekord University of Medical Sciences under (23 ± 2 °C) and 12/12 h light/dark cycle. Animals had unlimited access to standard food and water. Before the experiments, the mice were allowed to acclimate to the environment for one week. The general health of the mice was monitored during the experiments. All steps of experimentation were done in accordance with the protocols of the University and the Guide for the Care and Use of Laboratory Animals of National Institutes of Health (Ethics code: IR.SKUMS.-REC.1395, 193).

### 2.3. Experimentation and measurement of variables

In our experimental study, the animals were separated into 8 groups of 8. Groups consisted of group 1: received 0.5 ml of normal saline, group 2: received 6.1 mg/kg of CP, groups 3-5: received 50, 100, and 200 mg/kg of *A*. *atroviolaceum* extract alone, respectively, treatment groups (6-8): received 50, 100, and 200 mg/kg of *A*. *atroviolaceum* extract and 1.6 mg/kg of CP. All injections were performed intraperitoneally, once a day for 30 days [27]. Eventually, the mice were deep anesthetized using co-administration of ketamine (60 mg/kg, intraperitoneally) and xylazine (10 mg/kg, intraperitoneally) [28, 29] and the testicles and epididymis were surgically removed to perform examinations of interest.

Testicles and epididymis were separated from each other and the right and left testicles were separately weighed and placed in 10% formalin and placed in paraffin. Sections 5μ-thick were prepared and stained with haematoxylin and eosin (HE). The specimens were observed under an Olympus/3H light microscope. Then, seminiferous tubule diameter, lumen diameter, and epithelial thickness were determined at 100× magnification and the results were expressed in μm. By examining seminiferous tubule, the spermatogonia count, primary and round spermatocytes, and spermatozoa number were determined by using optical microscope [24, 30]. To determine the index of right and left testicles, the weight of each testicle was divided by the body's weight. The index is expressed as a percentage [31].

### 2.4. Statistical analysis

Information was evaluated by one-way ANOVA and Tukey's test in Prism 5 software. To determine the correlation, Pearson correlation coefficient was used. P < 0.05 was considered as Significance level.

## 3. Results

### 3.1. Influence of A. atroviolaceum extract on changes in lumen diameter

**Fig. 1:**
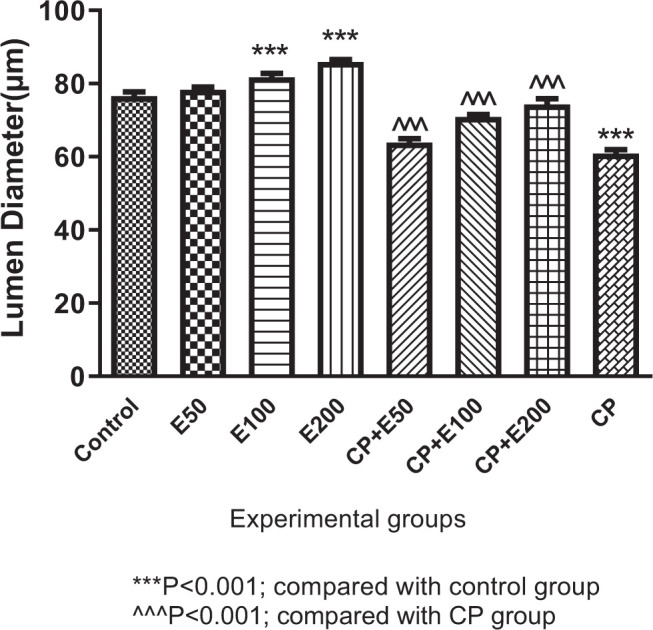
Effect of Allium atroviolaceum L. extract (E) on lumen diameter in cyclophosphamide (CP)-treated mice.

One-way ANOVA analysis of results presented that there was a significant difference in lumen diameter between different groups (P < 0.001). Tukey's post-test showed that the lumen diameter was significantly lower in the CP group than in the control group (P < 0.001). The groups treated with *A*. *atroviolaceum* extract at 100 and 200 mg/kg had significantly greater lumen diameter than the control group (*P* < 0.001). Lumen diameter was significantly higher in the groups treated with CP with extract at 50, 100, and 200 mg/kg than the group given CP alone (*P* < 0.001) (Fig. 1).

### 3.2. Influence of A. atroviolaceum extract on changes in testicular epithelial thickness

**Fig. 2:**
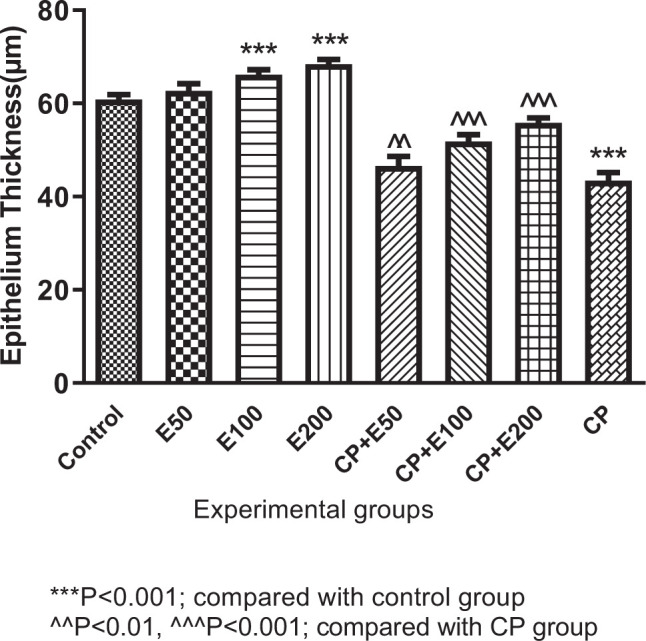
Effect of Allium atroviolaceum L. extract (E) on epithelial thickness in cyclophosphamide (CP)-treated mice.

One-way ANOVA analysis of results exposed a significant difference in epithelial thickness among different groups (*P* < 0.001). Results show that the epithelial thickness was significantly lower in the CP-treated group than in the group receiving normal saline (*P* < 0.001). Epithelial thickness was significantly greater in the groups treated with CP + extract at 50, 100 and 200 mg/kg than the CP-treated group (*P* < 0.001). In the groups that received 100 and 200 mg/kg of the extract, a significantly higher epithelial thickness was observed than in the group receiving normal saline (*P* < 0.001) (Fig. 2).

### 3.3. Effect A. atroviolaceum extract on changes in seminiferous tubule diameter

**Fig. 3:**
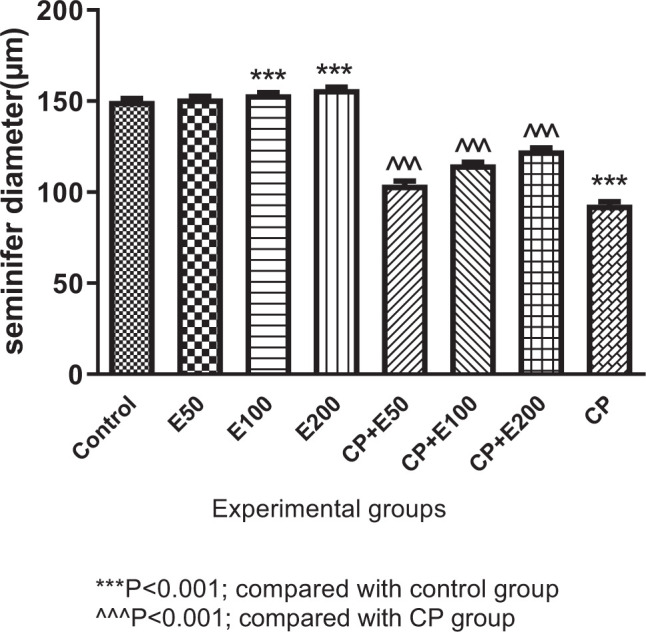
Effect of Allium atroviolaceum L. extract (E) on seminiferous tubule diameter in cyclophosphamide (CP)-treated mice.

One-way ANOVA analysis of results illustrated that there was a significant difference in seminiferous tubule diameter among different groups (*P* < 0.001). According to Fig. 3, Tukey's post-test showed that seminiferous tubule diameter was significantly different among different groups (*P* < 0.001). Seminiferous tubule diameter was significantly lower in the CP group than in the control group (*P* < 0.001). This variable was significantly higher in the treated groups with CP + extract at 50, 100 and 200 mg/kg than in the CP group (*P* < 0.001). In the extract groups, seminiferous tubule diameter was significantly higher than in the control group (*P* < 0.001).

### 3.4. Influence of A. atroviolaceum extract on changes in spermatozoa number

**Fig. 4:**
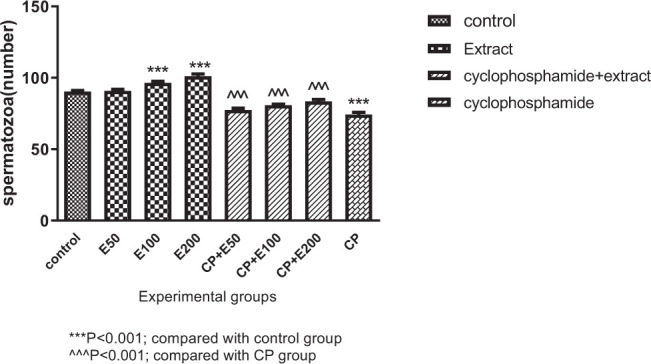
Effect of Allium atroviolaceum L. extract (E) on spermatozoa number in cyclophosphamide (CP)-treated mice.

According to Fig. 4, spermatozoa number was significantly lower in the CP group than in the group receiving normal saline (*P* < 0.001). Tukey's post-test showed that spermatozoa number in the groups receiving CP + extract at 50, 100, and 200 mg/kg was significantly higher when compared to the CP group (*P* < 0.001). In the treated groups with extract at 100 and 200 mg/kg, a significant higher number of spermatozoa was observed than in control group (*P* < 0.001).

### 3.5. Influence of A. atroviolaceum extract on changes in primary spermatocyte number

**Fig. 5:**
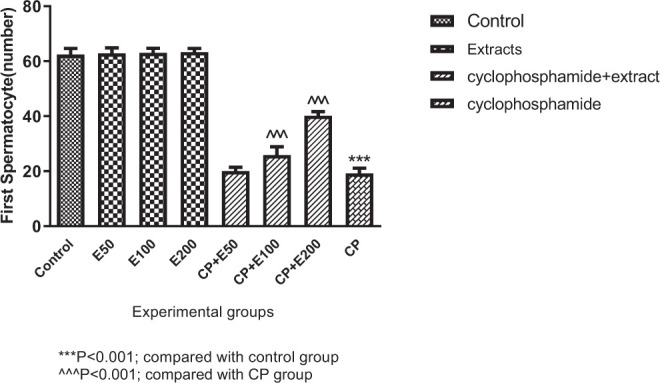
Effect of Allium atroviolaceum L. extract (E) on primary spermatocyte number in cyclophosphamide (CP)-treated mice.

According to Fig. 5, primary spermatocyte number was lower in a significant manner in the CP group than in the group receiving normal saline (*P* < 0.001). Primary spermatocyte number in the groups receiving CP extract at 100 and 200 mg/kg was significantly higher þ when compared to the CP group (*P* < 0.001).

### 3.6. Influence of A. atroviolaceum extract on changes in round spermatocyte number

**Fig. 6:**
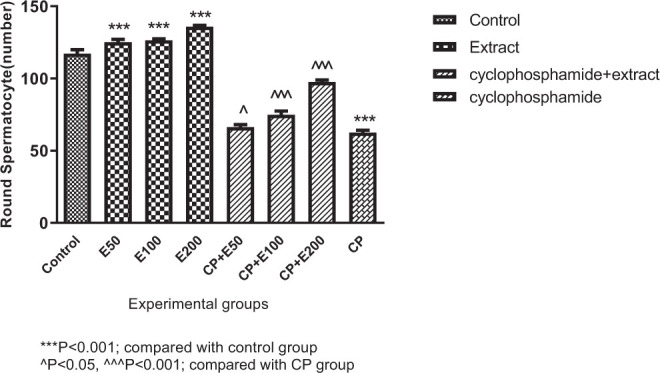
Effect of Allium atroviolaceum L. extract (E) on round spermatocyte number in cyclophosphamide (CP)-treated mice.

In this study, there was a significant difference in the number of round spermatocytes in different groups (*P* < 0.001). Tukey's post-test exhibited that the number of round spermatocytes in the CP group was significantly lower when contrasted to the group receiving normal saline (*P* < 0.001). The number of round spermatocytes in the treated group receiving CP + extract at 50 mg/kg and also in the treated group with CP + extract at 100 and 200 mg/kg was significantly greater in contrast to the CP group (*P* < 0.05 and *P* < 0.001, respectively). Additionally, round spermatocyte number in the groups receiving the extract at 50, 100, and 200 mg/kg was higher, significantly, compared to the CP-treated group (*P* < 0.001) (Fig. 6).

### 3.7. Influence of A. atroviolaceum extract on changes in spermatogonia number

**Fig. 7:**
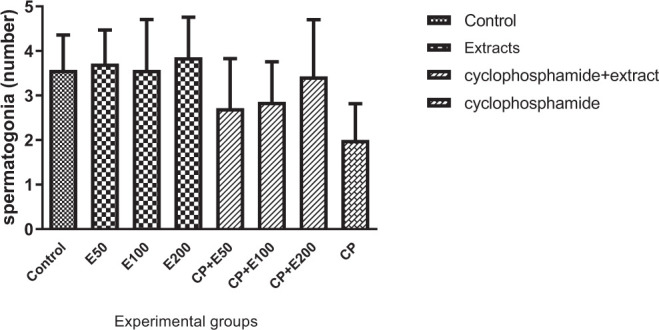
Effect of Allium atroviolaceum L. extract (E) on spermatogonia number in cyclophosphamide (CP)-treated mice.

As stated by the results of our study, the number of spermatogonia in the control and CP-treated groups and also extract groups and the groups receiving CP + extracts was not significant (Fig. 7).

### 3.8. Influence of A. atroviolaceum extract on changes in right and left testicle index

**Fig. 8:**
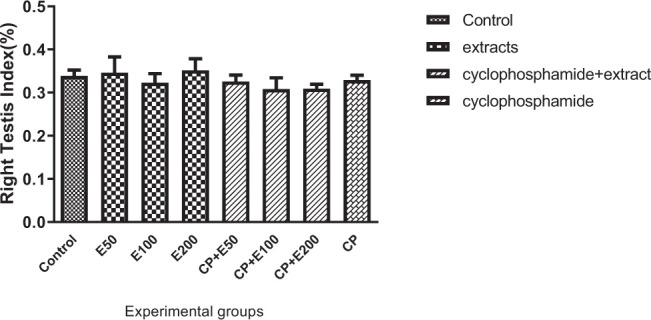
Effect of Allium atroviolaceum L. extract (E) on changes in right testicle index in cyclophosphamide (CP)-treated mice.

**Fig. 9:**
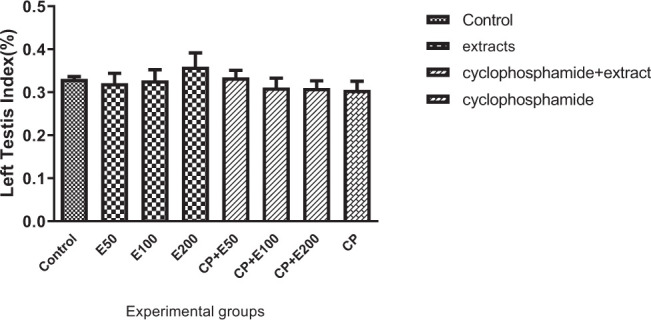
Effect of Allium atroviolaceum L. extract (E) on changes in left testicle index in cyclophosphamide (CP)-treated mice.

One-way ANOVA results showed that there was no significant difference in examined groups with respect to changes in right and left testicle index (*P* < 0.05). As stated by the results of our study, the changes in the right and left testicle index (Figs. 8 and 9, respectively) in the normal saline and CP groups and also the extract groups and the groups receiving CP + extracts at mg/kg were not significantly different.

## 4. Discussion

CP is widely used to treat cancer malignancies. Despite its therapeutic effects, CP may lead to an extensive variety of side effects, such as reproductive disorders, in consumers. Usually the therapeutic effects of CP are limited due to the confirmed testicular toxicity in various animal species [32]. Anomalies caused by CP are due to excessive oxidative stress. Therefore, the administration of antioxidants during CP therapy is necessary to reduce oxidative stress and to detoxify tissues [33]. This research evaluated the influence of *A*. *atroviolaceum* on testicular tissue in CP-treated mice. In the present study, the testicular morphology, including lumen diameter, epithelial thickness, and tubular diameter, were considerably lower in the CP group than in the controls. Additionally, the number of spermatozoa, primary spermatocytes, round spermatocytes, and spermatogonia in the CP group were considerably lower than that in the controls. CP had no significant effect on body weight and right and left testicles in mice. In general, the outcomes of this study exhibited that CP had no significant effect on the body weight and right and left testicles. Cyclophosphamide reduced the number of spermatozoa, primary spermatocyte, round spermatocyte and spermatogonia, and decreased lumen diameter, epithelial thickness, and tubular diameter.

In a study, CP treatment was associated with testicular tubule injury in mice, which is similar to the present research [32]. In the survey of Çeribaşi *et al*. (2010), CP treatment in mice was associated with a growth in the diameter of seminiferous tubules, a growth in germinal cell layer thickness, necrosis, damage and lack of maturity of germ cells, and testicular tissue atrophy, which is in agreement with the present review [34]. Another experimental study showed that the number of sperm cells, spermatids, and primary and secondary spermatocytes in the CP-exposed mice significantly decreased. In addition, CP caused atrophy of seminiferous tubules and decreased spermatogenic cells, which is similar to the outcomes of the present study [35].

In this research, *A*. *atroviolaceum* extract at 50, 100, and 200 mg/kg increased lumen diameter, epithelial thickness, tubular diameter, and spermatozoa and round spermatocyte number significantly contrasted with the controls. CP leads to reproductive toxicity by increasing the creation of reactive oxygen species, decreasing the glutathione content, and decreasing the activity of glutathione peroxidase [33, 36]. Glutathione peroxidase works as an antioxidant in protective spermatozoa in testicular and epididymal tissues and its decrease leads to infertility. This enzyme protects sperm against the free radicals by penetrating into the sperm plasma membrane, sperm nucleus, epididymal fluid, and epididymal region, and controls the evolution of the sperm [37, 38].

On the other hand, the ability of CP to produce free radicals, to peroxidize lipids, and to develop oxidative stress has been confirmed in rats [39, 40]. It has been stated that the administration of antioxidants along with CP reduces the toxicity of testicular tissue and the reproductive system significantly [33]. Oxidation-reduction balance is disrupted due to an increase in reactive oxygen species levels and free radicals and thus cellular activities, especially sperm production are disrupted as well. Antioxidants are scavengers that detoxify excessive ROS and have a serious role in maintaining oxidant and antioxidant balance in the body. [41, 42] Considering that the antioxidant activity of *A*. *atroviolaceum* extract has been confirmed *in vitro* and *in vivo* [43], it can be argued that the extract of this plant prevented damage to sex cells by decreasing the levels of free radicals and reactive oxygen species. A research managed by Nikravesh *et al*. (2010) to check the effect of *A*. *cepa* on testicular tissue in mice, showed that the extract of this plant increased spermato-genesis by influencing on the sperm ducts and cell proliferation in testicular tubules [44]. It has been argued that the presence of organic sulfur compounds in the plants of the *Allium* species significantly reduces the toxic influences of free radicals on the DNA of sperms by increasing glutathione levels and glutathione peroxidase activity. Organic sulfur compounds enhance the activity of antioxidant enzymes glutathione peroxidase and superoxide dismutase in different cells including hepatocytes, kidney cells, breast cells, and testicular cells, and protect cells via influencing peroxide and oxidative forms [45]. Flavonoids as major secondary metabolites in *A*. *atroviolaceum* are phenolic com-pounds which display a variety of biological activities, such as antioxidant, anti-inflammatory, blood lipid-lowering and anticarcinogenic activities. In addition to possessing in vitro antioxidant activity, they may have beneficial effects on capillary permeability and blood flow [26]. They also exhibit anti-allergy and anti-inflammatory benefits from in vitro studies. Several studies have reported that antioxidants in diet can protect sperm DNA from free radicals and increase blood testis barrier stability [24, 46, 47]. So presence of sulfur and flavonoid compounds in *A. atroviolaceum* may exhibit protective effects on reproductive system in synergy.

## 5. Conclusion

Research confirmed the protective effect of *A*. *atroviolaceum* extract in contrast to testicular tissue damage in CP-treated male mice. Therefore, this plant can be used to prevent or reduce the harm caused by CP to the reproductive system in patients treated with this drug. Although the function of this extract still needs to be further investigated in human studies. Future studies can focus on the isolation of the main constituents affecting the reproductive system in this plant.

## Supplementary materials
