# Supplementary material for: Effect of hydroalcoholic Allium atroviolaceum L. on the pathology of testicular tissue in cyclophosphamide-treated mice
Source: Biomedicine (Taipei). 2020 Sep 1;10(3):25–32. doi: 10.37796/2211-8039.1026 (PMC7721469; doi:10.37796/2211-8039.1026)
Supplement: Supplementary file 2 [file bmed-10-03-025-s002.docx]

Dear Editor;

Thanks for your consideration, all comments were checked and replied as comments on the pdf file as well as all queries were replied as below. Please see the attached file.

Regards,

**Q1:**

ok, all of them were checked.

**Q2:**

**Acknowledgements**

The financial support of the Deputy of Research and Technology of Shahrekord University of Medical Sciences is appreciated (Grant No: 2259). The authors of this study appreciate the Medical Plants Research Center Shahrekord, Iran and all participated in this study.

**Funding**      
 This study was funded by the Research and Technology Deputy of Shahrekord University of Medical Sciences (grant number 2259).

**Q3:**

Yoshimura Y, Subedi H, Nishibori K. Impact of oxidative stress and supplementation with vitamins E and C on testes morphology in rats. J Reprod Dev 2006; 52(2):203-209

**Q4:**

ok, all of them were checked.
