# Supplementary material for: Effect of hydroalcoholic Allium atroviolaceum L. on the pathology of testicular tissue in cyclophosphamide-treated mice
Source: Biomedicine (Taipei). 2020 Sep 1;10(3):25–32. doi: 10.37796/2211-8039.1026 (PMC7721469; doi:10.37796/2211-8039.1026)
Supplement: Supplementary file 4 [file bmed-10-03-025-s004.docx]

Dear Editor;

Thanks for your consideration. Now the range of pages is correct.

Regards,
